# Design of short-sequenced collagen type III-derived peptides with GPR56 dependent biological activity

**DOI:** 10.1042/BSR20250320

**Published:** 2026-11-03

**Authors:** Israa Mohammed Al-Amily, Fariborz Parandeh, Pontus Dunér, Marie Sjögren, Albert Salehi

**Affiliations:** 1 Department of Clinical Science, Malmö, Division of Islet Cell Physiology, University of Lund, Sweden; 2 Department of Clinical Science, Malmö, Division of Experimental Cardiovascular Research, University of Lund, Sweden

**Keywords:** DNA alignment, GPCRs, intracellular signaling, Pancreatic islets, peptides, qPCR

## Abstract

We have previously shown that activation of G protein-coupled receptor 56 (GPR56) by its endogenous ligand collagen type III (Coll III) improves pancreatic β-cell function and potentiates glucose-stimulated insulin secretion (GSIS). In the present study, alignment of Coll III and GPR56 genomic DNA revealed three complementary nucleotide regions, enabling the design of three short peptides (NQGCKL·NH_2_, H·LLSSR·NH_2_, and NH_2_·GYYCG·COOH). The peptides in double or triple combinations markedly increased cellular cAMP levels, enhanced GSIS, and improved cell viability both in human (EndoC βH1 cells) and rat pancreatic β-cell lines (INS-1 832/13 cells). These effects were markedly absent in GPR56-knockdown cells, supporting receptor-dependent specificity. The peptide mixtures also counteracted cytokine- and glucotoxicity-induced cellular stress, restoring cAMP generation and insulin secretion. Comparable effects were not observed with dapagliflozin, while glibenclamide decreased cell viability under similar conditions. In hepatic cell lines (HepG2 and Hep1–6), peptide combinations enhanced cell survival in a GPR56-dependent manner, consistent with a broader role of GPR56 in metabolic regulation. Collectively, the data identify three short sequence collagen III-derived peptides whose biological effects are dependent on GPR56 expression and support their further investigation as tools for studying GPR56-associated cellular signaling for the future *in vivo* validation.

## Introduction

Metabolic syndrome leads to type 2 diabetes (T2D) development through progressive β-cell dysfunction and increased peripheral insulin resistance [1–3]. While β-cells initially compensate for the insulin resistance by increasing their secretory ability, chronic stress causes an increased demand for insulin, which eventually induces β-cell stress leading to impaired insulin secretion and decreased cellular viability [4–6]. Identifying mechanisms that preserve β-cell function and survival during pathophysiological conditions is therefore crucial for developing preventive therapies.

G-protein coupled receptors (GPCRs) constitute the largest family of cell-surface receptors, regulating numerous physiological processes in the body and thus constituting a rich source of targets for pharmacological prevention of various human diseases, including metabolic syndrome and T2D [7–9]. We previously identified G protein-coupled receptor 56 (GPR56; ADGRG1) as one of the most abundantly expressed GPCRs in human pancreatic islets, liver, and subcutaneous adipose tissue [7,8,10]. Activation of GPR56 by its endogenous ligand, collagen type III (Coll III), enhances β-cell viability and insulin secretion [10–12]. Because Coll III is a large extracellular matrix protein [12–14], designing smaller peptides that mimic the collagen effects by selectively activating GPR56 could offer new pharmacological tools. Functionally, as previously demonstrated by our group and others, GPR56 activation primarily engages Gαs to stimulate adenylate cyclase in pancreatic β-cells, increasing cellular cAMP levels [10,11,15]. Thus, the analysis of cellular cAMP could serve as a signaling when studying the effect of GPR56 agonists on pancreatic β-cells. Notably, an increase in intracellular calcium concentration [Ca^2+^]_i_ via Gα_q_ has also been reported in cells overexpressing Gαq [12]. However, this was not clearly linked to Gα_q_ since it, in contrast with ATP-induced [Ca^2+^]_i_ rise, was abolished in the absence of extracellular Ca^2+^ [12,15], suggesting context-dependent signaling versatility and less suitability for signal analysis.

### Hypothesis

Although several established strategies for peptide design such as structure-based modeling, computational modeling, and docking or experimental screening are available, these approaches are often time- and resource-intensive [16–18]. We therefore hypothesize that a simplified, DNA nucleotide sequence-guided strategy could be exploited to identify candidate peptide motifs involved in the interaction of a GPCR with its endogenous peptide ligand. Specifically, we hypothesize that alignment of the Coll III DNA strand with the GPR56 DNA strand may reveal regions of complementary or related nucleotide patterns that, upon transcription and translation, would give rise to detection of short peptide sequences with potential functional relevance. While protein–protein interactions are not governed by nucleotide complementarity *per se*, this alignment-based strategy may serve as a heuristic tool to identify amino acid motifs enriched in regions involved in ligand-receptor interaction. Using this approach, we generated a set of three short peptides predicted from aligned Coll III-GPR56 DNA complementary regions. The possible effect of the synthesized peptides was evaluated in β-cells and hepatocytes under normal, glucotoxicity, and inflammatory conditions in both scrambled control cells and GPR56 knockdown (GPR56-KD) models. We found that the peptides in double and triple combinations positively modulate β-cell function.

## Materials and methods

### Reagents

Fatty acid-free bovine serum albumin (Boehringer, Germany), the insulin ELISA kit (Mercordia, Sweden), primers, and quantitative polymerase chain reaction (qPCR) materials were from Applied Biosystems (Waltham, Massachusetts, U.S.A.). The COL III-based short-sequenced peptides used in the experiments were produced by Red Glead Discovery, with a purity of 99.5% (Lund, Sweden). All other chemicals were from Merck AG (Darmstadt, Germany) or Sigma (Saint Louis, MO, U.S.A.).

### Collagen type III-based peptide design and sequence selection

The coding DNA sequences of human COL III and GPR56 (ADGRG1) were aligned to identify regions displaying complementary or related nucleotide sequence patterns. Candidate regions were selected based on the observed sequence correspondence and interestingly showed localization within the extracellular region of GPR56. The corresponding nucleotide sequences were translated to identify short peptide sequences derived from COL III. The candidate peptides were subsequently selected for experimental synthesis based on their correspondence with regions located within the extracellular N-terminal/GAIN region of GPR56. The peptides were custom synthesized by Red Glead Discovery (Lund, Sweden) at a reported purity of 99.5%.

### Cell culture

#### INS-1 832/13 cells

INS-1 832/13 cells (kindly donated by Dr C.B. Newgaard, Duke University, U.S.A.) were cultured in RPMI-1640 containing 11.1 mM D-glucose and supplemented with 10% fetal bovine serum, 100 U/ml penicillin (Gibco, BRL, Gaithersburg, MD), 100 μg/ml streptomycin (PAA Laboratories, Toronto, Ontario, Canada), 10 mM N-2 hydroxyethylpiperazine-N′-2-ethanesulfonic acid (HEPES), 2 mM glutamine, 1 mM sodium pyruvate, and 50 μM β-mercaptoethanol (Sigma−Aldrich, Saint Louis, MO, U.S.A.), at 37°C in a humidified atmosphere containing 95% air and 5% CO_2_. To assess the effects of elevated glucose (glucotoxicity), cells were treated with 25 mM D-glucose (25G) for 72 h. Controls were maintained in 5 mM glucose (5G) media. For GPR56 knockdown by siRNA, INS1 832/13 cells were cultured to 75% confluency and then subjected to *GPR56*-KD as previously described [10,11]. The siRNAs used for *Gpr56*-KD are shown in Supplementary Table S1. Each treatment was carried out in three biological replicates, along with scrambled controls. The extent of GPR56-KD was quantitatively determined by qPCR (Supplementary Figure S1). Thereafter, the cells were washed and cultured for 6 h in a standard RPMI medium (recovery period) before being cultured in RPMI-1640 with 5 or 25 mM glucose (5G or 25G) in the presence or absence of agents for 72 h (Figure 1) or being subjected to incubation with indicated substances.

**Figure 1:**
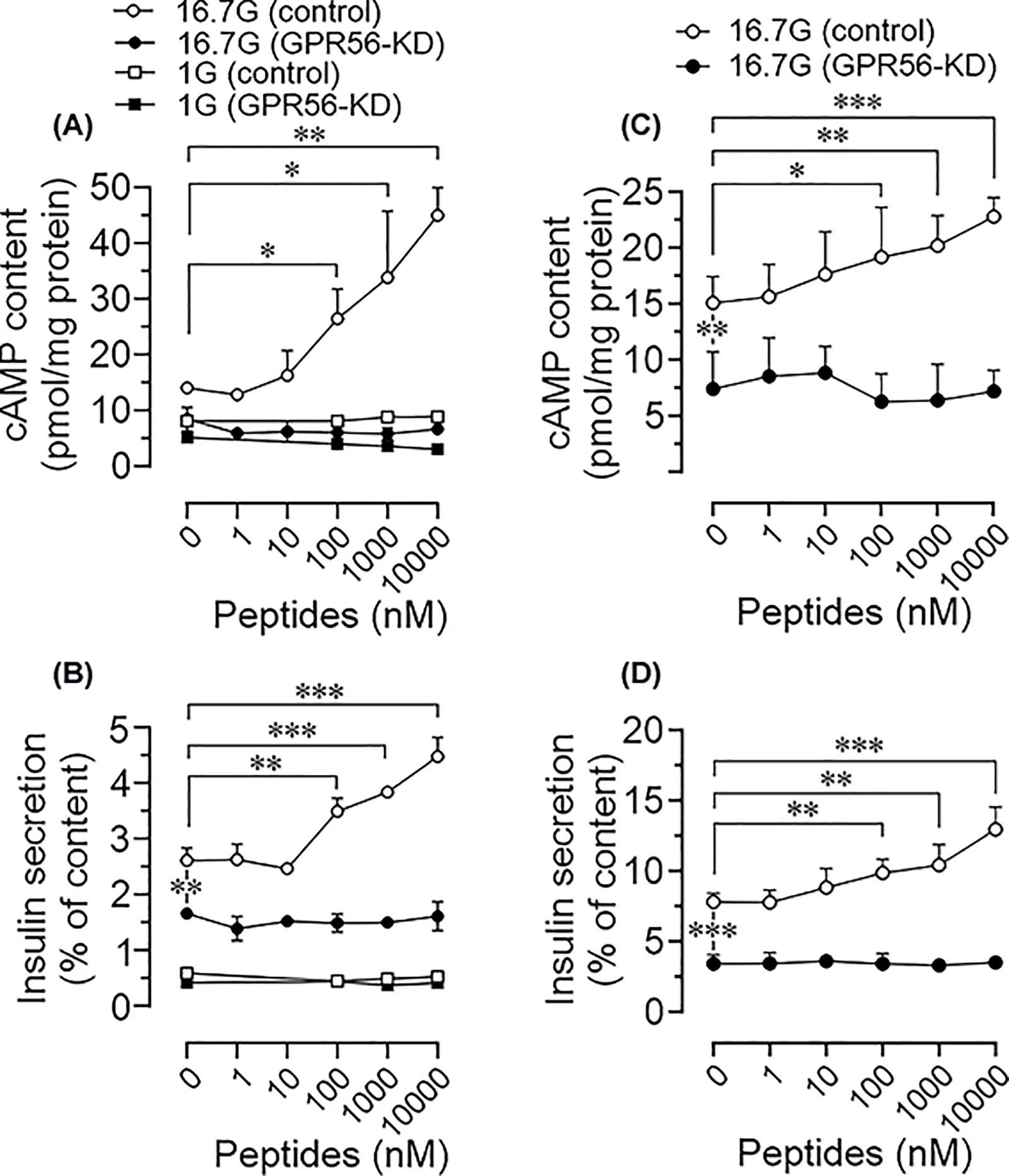
Concentration-dependent effect of the peptide mixture on cAMP content and insulin secretion Concentration-dependent effect of the peptide mixture on cAMP content (**A**) and insulin secretion (**B**) in scrambled control and GPR56-KD EndoC βH1 cells incubated at 1 mM or 16.7 mM glucose for 60 min. Results are mean ± SD for *n =* 4 different passages. *P<0.05, **P<0.01, ***P<0.001 (unpaired Student’s *t*-test analysis). Concentration-dependent effect of the peptide mixture on glucose-stimulated (16.7 mM) cAMP generation (**C**) and insulin secretion (**D**) in scrambled control and GPR56-KD INS-1 832/13 cells incubated at 16.7 mM glucose for 60 min. Data represent mean ± SD for *n =* 5. *P<0.05, **P<0.01, ***P<0.001 (unpaired Student’s *t*-test analysis between indicated groups).

#### EndoC βH1 cells

The human clonal β-cell line EndoC-βH1 (EndoCells, Paris, France) was seeded in a 24-well plate at a density of 1.8 × 10^5^ cells/well and maintained in DMEM culture medium (4.5 mM glucose), 2% BSA fraction V (Roche, Basel, Switzerland), 10 mM nicotinamide (Merck, Darmstadt, Germany), 50 μM 2-mercaptoethanol, 5.5 μg/ml transferrin, 6.7 ng/ml sodium selenite (Sigma), 100 U/ml penicillin, and 100 μg/ml streptomycin (PAA Laboratories, Toronto, Ontario, Canada) as described [19]. For measurement of insulin secretion, the cells were cultured for 12 h in the culture medium containing 2.8 mM glucose before incubation at 1 or 16.7 mM glucose.

#### Min6 cells

Clonal mouse insulin-secreting Min6 cells were seeded at 1 × 10^4^ cells/well into 24 well plates in DMEM Dulbecco’s modified Eagle’s medium (DMEM+GultaMax™-l, Gibco, U.S.A.) containing 4.5 g/l glucose supplemented with 15% fetal calf serum, 50 mg/1 streptomycin (Gibco), 75 mg/1 penicillin sulphate (Gibco), and 5 μl/ml β-mercaptoethanol (Sigma). The cells were then transfected with lentiviral particles targeting Gpr56. After transfection, the cells were washed with PBS (×3) and subjected to total RNA extraction for qPCR analysis. Validation of Gpr56-KD was determined by qPCR (Supplementary Figure S1).

### Knockdown of GPR56 in mouse Hep1–6, INS-1 832/13, and human EndoC βH1 or Hep2-G cells

GPR56-KD in mouse Hep1–6 cells was performed using a cocktail of three different lentivirus-delivered shRNAs targeting the GPR56 gene (Santa Cruz, CA, U.S.A.) as described previously [10,11]. For down-regulation of GPR56 in INS-1 832/13 cells and EndoC βH1 cells, siRNA from ThermoFisher Scientific (Wilmington, DE, U.S.A.), with an appropriate scrambled control, was used according to the manufacturer's recommendations. Validation of GPR56-KD was determined by qPCR (shown in Supplementary Figure S1) and by confocal microscopy as reported previously [10,11]. The siRNAs or shRNAs used for GPR56-KD are shown in Supplementary Table S1.

### Quantitative polymerase chain reaction

Total RNA was extracted from Min6, INS1 832/13, and EndoC-βH1 cells using RNAeasy (Qiagen, Hilden, Germany) before complementary DNA was synthesized using SuperScript (Invitrogen, Carlsbad, CA, U.S.A.), according to the manufacturer's protocol. Concentration and purity of total RNA was measured with a NanoDrop ND-1000 spectrophotometer (A260/A280>1.9 and A260/A230>1.4) (NanoDrop Technologies, Wilmington, DE), and an RNA quality indicator higher than 8.0 (Experion Automated Electrophoresis, Bio-Rad, U.S.A.) was considered to be a high-quality total RNA preparation. TaqMan mastermix from Applied Biosystems (Foster City, CA, U.S.A.) was used for qPCR and performed following the manufacturer’s protocol and was run in a 7900HT Fast Real-Time System (Applied Biosystems). The qPCR was carried out as follows: 50°C for 2 min, 95°C for 10 min, 40 cycles of 95°C for 15 s, and 60°C for 1 min. Changes in gene expression were calculated using the ΔΔCt method with a fold change cut-off at ≥1.5 and *P<*0.05 considered significant. All samples were run in duplicate, and relevant negative controls were run on each plate. qPCR results were normalized to housekeeping genes (PPIA or HPRT). Primer information used in the qPCR assays is provided in Supplementary Table S2.

### cAMP determination

The cAMP content in the cell lysate was measured using a direct cAMP ELISA kit (Enzo Life Sciences, Farmingdale, NY, U.S.A.) according to the manufacturer's instructions, and the values were related to protein content. The protein concentrations of the cell lysates were measured by a BCA kit (Thermo Fisher Scientific, Wilmington, DE, U.S.A.) as reported previously [20].

### Insulin secretion

For functional studies, after recovery, the siRNA-treated INS-1 832/13 or EndoC βH1 cells were washed and pre-incubated for 120 min at 37°C in SAB buffer, pH 7.4, supplemented with 10 mM HEPES, 0.1% bovine serum albumin, and 2.8 mM glucose. After pre-incubation, the buffer was changed, and INS-1 832/13 or EndoC βH1 cells were incubated at 1 or 16.7 mM glucose with indicated agents for 60 min at 37° C. Immediately after incubation, an aliquot of the medium was removed and frozen for subsequent assay of insulin. The secreted insulin in the incubation medium as well as the insulin content of the cells was measured by ELISA (Mercodia, Sweden). The data was presented as secreted insulin/insulin content (% of the content). The cells were then washed with PBS and stored in 100 mM HCl containing IBMX (100 μM) for subsequent cAMP analysis.

### Statistics

The results are means ± SD for the indicated number of experiments. The random differences and significance in normally distributed data sets (Shapiro–Wilk test) were then analyzed by Student’s *t*-test when comparing two groups, i.e. a treated group versus basal control. When three or more groups were compared, statistical significance was assessed by one-way ANOVA followed by the Tukey–Kramer multiple-comparisons test. *P*-value <0.05 was considered significant.

## Results

### Design of peptides based on human collagen type III and GPR56 gene structures and their possible effect on β-cell function

The DNA sequence and protein structure of both GPR56 and its naturally occurring ligand, Coll III, are well established and publicly available (https://alphafold.ebi.ac.uk; UniProt). In the first stage of study, we exploited a novel, sequence-guided strategy to derive short peptide motifs from the alignment of the coding DNA sequence of GPR56 with the coding DNA sequence of Coll III to identify regions of potential complementarity or sequence correspondence. This alignment revealed two distinct regions in GPR56 exhibiting complementary nucleotide patterns to Coll III, as well as a third region of interest that, although not strictly complementary, displays features suggestive of potential functional relevance (Tables 1 and 2). The identified regions on GPR56 and Coll III were conceptually transcribed into mRNA sequences, allowing the corresponding amino acids of the translated peptide sequences to be inferred. The analysis thus showed on the GPR56 at amino acid positions 225–231 (MVSFEED) and 338-341 (VTLQ) on the extracellular N-terminal (PLL/GAIN) domain with the reflective complementary amino acid sequences (NQGCK-NH_2_ and H-LLSSR-NH_2_, respectively) on Coll III (Supplementary Figure S2). The analysis also showed an additional region at amino acid positions 88–92 on GPR56 (YHFCL), which was translated to the corresponding peptide (NH_2_-GYYCG-COOH) (Supplementary Figure S2). Based on these amino acid combinations, three short peptides were custom synthesized with 99.5% purity (see the ‘Reagents’ section) for further functional testing.

**Table 1:** Experimentally identified Coll III-derived peptides after DNA alignment

Peptide	Coding DNA	Transcript	Exon	Amino acid position	Protein domain
NQGCKL-NH_2_	AACCAAGGATGCAAATTG	NM_000090	48	1284–1289	C-propeptide
H-LLSSR-NH_2_	CTCCTCTCCAGTAGA	NM_000090	50	1359–1363	C-propeptide
H-GYYCG-COOH	GGCTACTACTGCGGC	NM_000090	engineered sequence	1420–1422	C-propeptide

The peptide amino acid sequences, their localization within the canonical human Coll III protein, including the corresponding coding DNA sequences, reference transcript, exon localization, amino acid positions, and protein domain assignment. The red-colored amino acid shows modification of the naturally occurring one to reinforce the peptide and its stability.

**Table 2:** Experimentally identified GPR56-derived peptides after DNA alignment

Peptide	Coding DNA	Transcript	Exon	Amino acid position	Protein domain
MVSFEED	ATGGTGAGCTTCGAGGAGGAC	NM_005682	2	225–231	Signal peptide
VTLQ	GTGACTCTGCAG	NM_005682	8	338–341	GAIN domain
YHFCL	TACCACTTCTGCCTG	NM_005682	3	88–92	GAIN domain

The amino acid sequences and their localization within the canonical human GPR56 protein, including the corresponding coding DNA sequences, reference transcript, exon localization, amino acid positions, and protein domain assignment.

### Peptide effects on cAMP and insulin secretion in β-cell models

To assess the biological activity of the peptides, we first tested their ability to modulate β-cell responses in a GPR56-dependent manner. Human EndoC-βH1 and rat INS-1 832/13 cells were used. Scrambled control and GPR56-KD EndoC-βH1 cells were preincubated at 2.8 mM glucose for 120 min, followed by incubation with 1 or 16.7 mM glucose with increasing peptide concentrations (0–10000 nM) for 60 min.

As shown in Figure 1A, high glucose increased cAMP content in EndoC-βH1 cells in parallel with glucose-stimulated insulin secretion (GSIS) (Figure 1B). The peptide mixture did not affect cAMP content (Figure 1A) or insulin release at basal glucose (1 mM) (Figure 2B). However, at high glucose (16.7 mM), the peptide mixture increased cellular cAMP levels and potentiated GSIS in a concentration-dependent manner, with significant effects observed from 100 nM onwards. These effects were markedly reduced in GPR56-KD cells, supporting a receptor-dependent mechanism.

**Figure 2:**
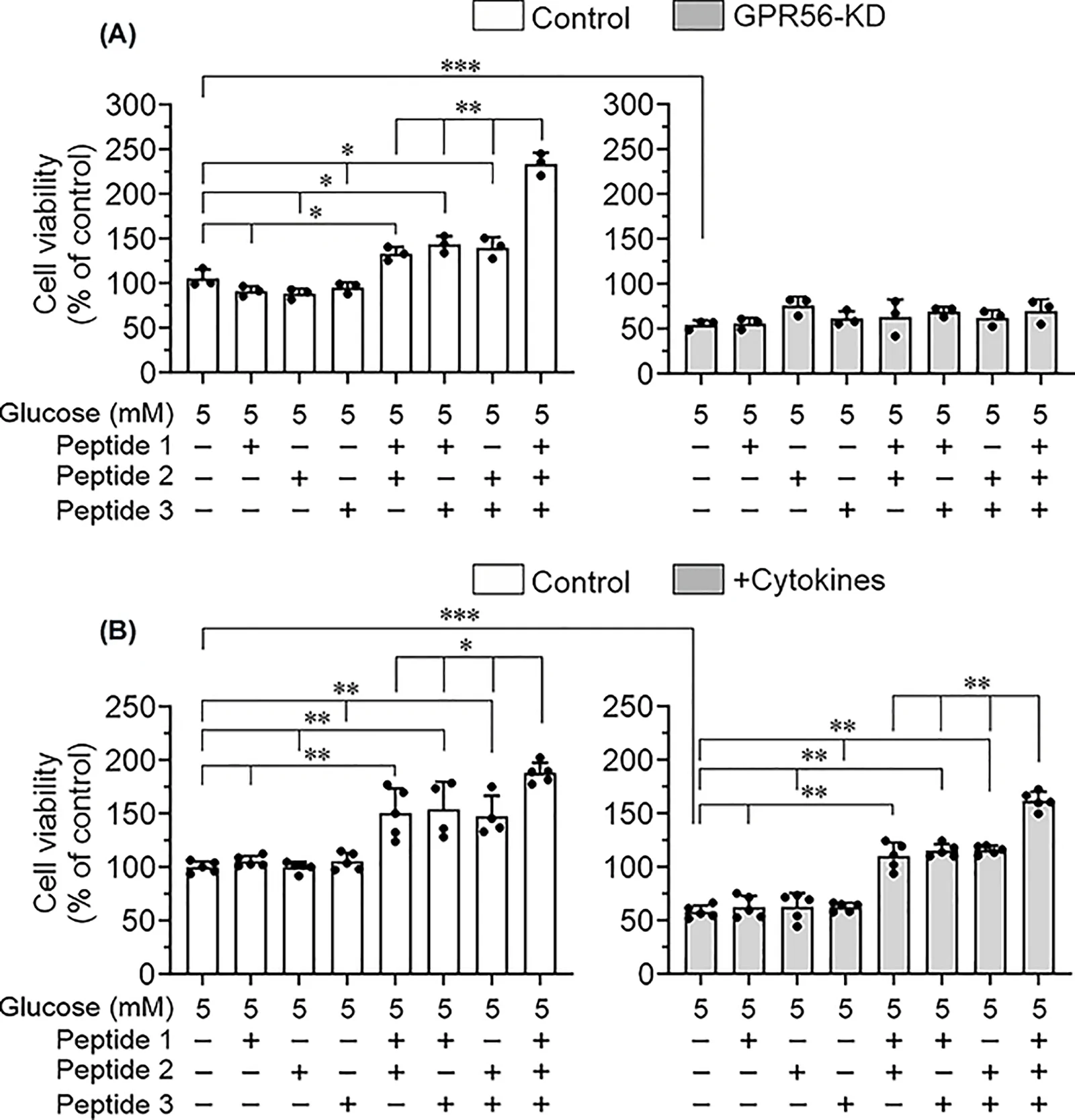
The effect of peptides alone or in double and triple combination on β-cell viability (**A**) Comparative effects of the three different peptides (10 μM) alone or in double (peptide 1+2, 1+3, and 2+3) and triple (peptide 1+2+3) combinations on cell viability in scrambled control and GPR56-KD INS-1 832/13 cells cultured for 24 h. Thereafter, the cell viability (reductive capacity) was measured by MTS. The results are mean ± SD for *n =* 3 experiments. *P<0.05, **P<0.01, ***P<0.001 (unpaired Student’s *t-*test analysis). (**B**) INS-1 832/13 cells were cultured with or without a cytokine cocktail (IL-1β, 1 ng/ml; TNFα, 50 ng/ml; and IFNγ, 50 ng/ml) in the presence or absence of peptides (10 μM each) alone or in double (peptide 1+2, 1+3, and 2+3) and triple (peptide 1+2+3) combinations for 24 h. Thereafter, the cell viability (reductive capacity) was measured by MTS. The data are mean ± SD from *n =* 5 different experiments. *P<0.05, **P<0.01, ***P<0.001 (unpaired Student’s *t*-test analysis between two indicated groups).

Similar dose-dependent effects of the peptide mixture on cAMP and GSIS were observed in INS-1 832/13 cells (Figure 1C,D). In this model, GPR56-KD also led to reduced cAMP levels and impaired insulin secretion in response to high glucose, suggesting an intrinsic activity of GPR56 in regulating β-cell responsiveness. The efficiency of GPR56 knockdown in cell lines is shown in Supplementary Figure S1.

### Effects of individual versus combined peptides on cell viability

We next assessed whether peptides exerted effects individually or only in combination. Scrambled control and GPR56-KD INS-1 cells were cultured at 5 mM glucose in the presence of 10 μM peptides, either alone or in double or triple combinations. Cell viability was measured using MTS assays.

As shown in Figure 2, none of the individual peptides significantly affected cell viability. In contrast, double combinations (1+2, 1+3, and 2+3) significantly increased cell viability, and the triple combination of the peptides (1+2+3) even produced the most substantial effect (Figure 2).

### Peptide mixture protects β-cells from cytokine-induced cytotoxicity

Pancreatic β-cell culture with proinflammatory cytokines (IL-1β, TNF-α, and IFN-γ) is known to impair GSIS and reduce viability [21–23]. To test whether our peptides could counteract these effects, INS-1 832/13 cells were cultured for 24 h with cytokines (IL-1β: 1 ng/ml, TNF-α: 50 ng/ml, and IFN-γ: 50 ng/ml) in the absence or presence of peptides.

Cytokine exposure markedly reduced cell viability compared with controls (Figure 2B). Individual peptides did not significantly prevent cytokine-induced damage. However, double peptide combinations restored cell viability to near-control levels, and the triple peptide combination was even more effective in counteracting cytokine toxicity (Figure 2B).

### Peptides restore β-cell function under glucotoxicity conditions

Chronic exposure of β-cells to high glucose induces oxidative stress, ER stress, and mitochondrial dysfunction, leading to impaired GSIS [24–27]. Since incretin hormones (GLP-1, GIP, and PACAP38) counteract such stress partly by stimulating cAMP generation [3,28–33], we tested whether our peptide mixture could provide similar benefits.

INS-1 832/13 cells were cultured at 5 or 25 mM glucose for 72 h in the presence or absence of peptide mixture, followed by incubation with 1 or 16.7 mM glucose. At 5 mM glucose, peptide-treated cells showed elevated basal cAMP (1 mM glucose) and potentiated GSIS-associated cAMP production (16.7 mM glucose) (Figure 3). At 25 mM glucose, cells showed reduced cAMP levels and GSIS, consistent with negative effects of glucotoxicity. Notably, co-culture with peptide mixture (consisting of 10 μM of each peptide) restored both cAMP and GSIS to levels comparable to controls (Figure 3A,B).

**Figure 3:**
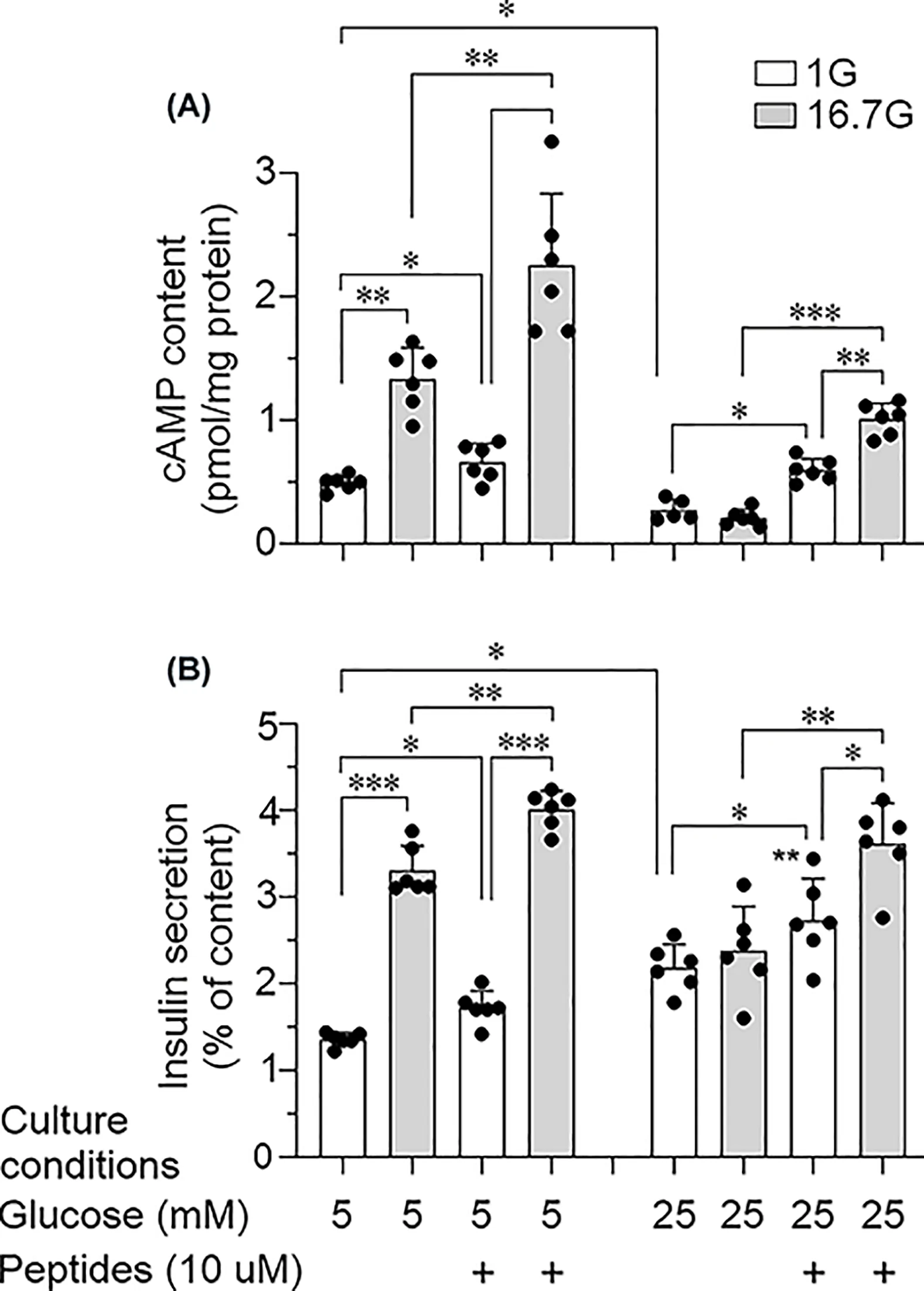
Prevention of glucotoxicity-induced β-cell dysfunction by the peptide mixture INS-1 832/13 cells were cultured at 5 mM glucose ± peptide mixture (10 μM) or 25 mM glucose ± peptide mixture (10 μM of each peptide) for 72 h. Thereafter they were incubated at 1 or 16.7 mM glucose for 60 min, and cAMP (**A**) and GSIS (**B**) were recorded. Prior culture with the peptides increased basal cAMP at 1 mM glucose and also potentiated glucose-stimulated (16.7 mM) cAMP generation, which was reflected in an increased insulin secretory capacity (B). Prior culture of INS-1 832/13 cells at high glucose (25 mM) decreased cAMP content at basal (1 mM glucose) and abolished glucose-stimulated cAMP generation (A), which was reflected in a diminished GSIS (B). The presence of peptide mixture during the culture period prevented the detrimental effect of high (25 mM) glucose, as it is reflected in an increased glucose-stimulated cAMP production accompanied by an increased GSIS. Data represent mean ± SD for *n =* 6 observations performed at different occasions. *P<0.05, **P<0.01, ***P<0.001 (unpaired Student’s *t*-test analysis between two indicated groups).

### Comparison with dapagliflozin and glibenclamide

To benchmark peptide efficacy, we compared their effects with two established antidiabetic drugs, dapagliflozin and glibenclamide, in EndoC-βH1 cells. Cells were cultured for 72 h at 5 or 25 mM glucose with a mixture of peptides (10 μM of each), dapagliflozin (10 μM), or glibenclamide (5 μM).

At 5 mM glucose, dapagliflozin did not affect cell viability, peptides increased viability, while glibenclamide decreased it (Figure 4A). At 25 mM glucose, cell viability was reduced; peptides prevented this reduction, while dapagliflozin had no effect, and glibenclamide tended to worsen viability.

**Figure 4:**
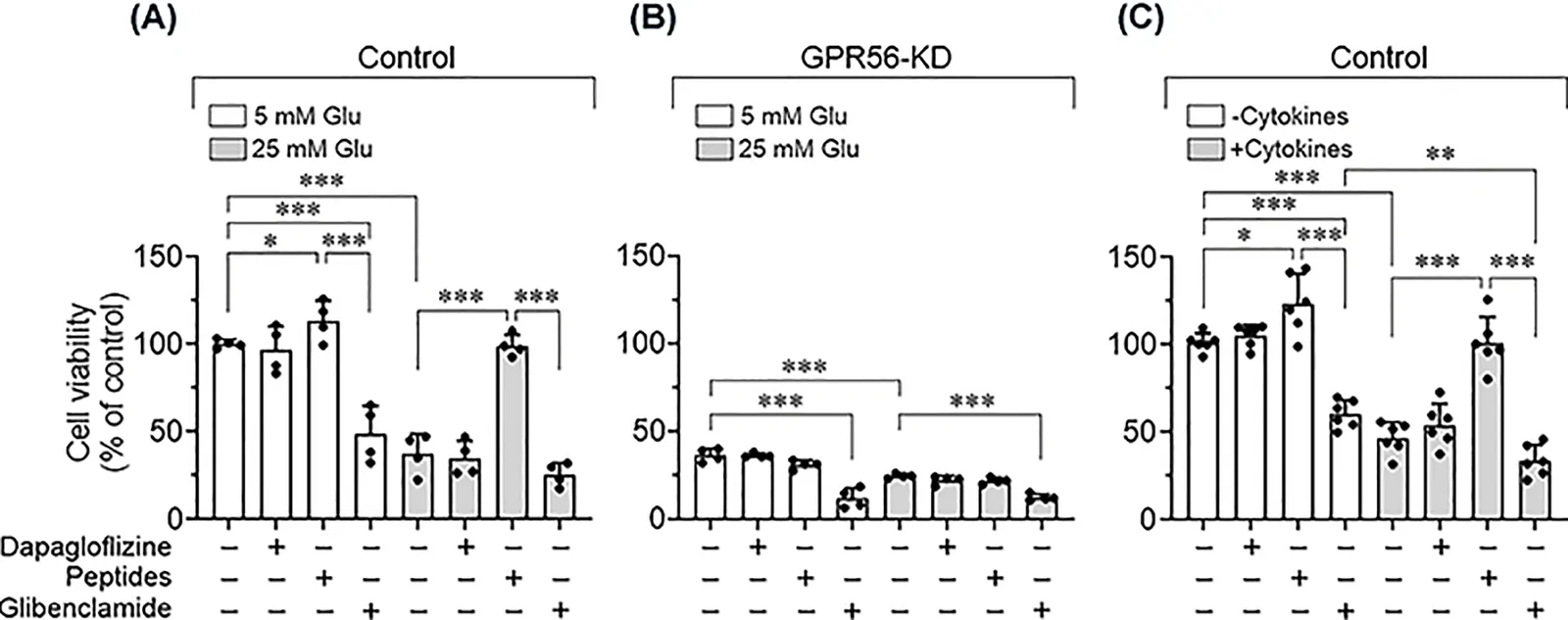
Comparing the effect of peptides with dapagliflozin and glibenclamide on cell viability at different experimental conditions Scrambled control and GPR56-KD EndoC βH1 cells were cultured at 5 mM or 25 mM glucose in the presence or absence of dapagliflozin (10 μM), peptide mixture (10 μM of each peptide), or glibenclamide (5 μM) for 72 h. Thereafter cell viability (reductive capacity) was measured by MTS (see the ‘Materials and methods’ section). Dapagliflozin neither have any effect at 5 mM nor could prevent decreased cell viability at 25 mM glucose, while peptide mixture modestly increased cell viability at 5 mM glucose and prevented the high glucose-induced (25 mM) diminished cell viability and restored it to almost the same levels as in the 5 mM glucose control. Glibenclamide had a deteriorating effect on cell viability at basal (5 mM glucose) and further intensified the high glucose-induced decrease in cell viability (**A**). GPR56-KD decreased cell viability at 5 mM glucose and deteriorated high glucose-induced (25 mM) decrease in cell viability. Dapagliflozin neither affect cell viability at basal (5 mM glucose) nor at high glucose (25 mM) in GPR56-KD EndoC βH1 cells. In contrast with scrambled controls, peptide mixture did not have any improvement of cell viability, neither at 5 mM nor at 25 mM glucose in GPR56-KD INS-1 832/13 cells. The deteriorating effect of glibenclamide was more pronounced in GPR56-KD cells both at 5 mM and at 25 mM glucose (**B**). Data are mean ± SD for *n =* 4 observations performed at different occasions. *P<0.05, **P<0.01, ***P<0.001. Culture of EndoC βH1 cells with a cytokine cocktail (IL-1β, 1 ng/ml; TNFα, 50 ng/ml; and IFNγ, 50 ng/ml) for 24 h markedly decreased cell viability as measured by MTS (**C**). Shown is the effect of dapagliflozin (10 μM), peptide mixture (10 μM), or glibenclamide (5 μM) on cytokine-induced reduction in cell viability when they were present during the culture period. The results represent mean ± SD for *n =* 6 observations performed at different occasions. *P<0.05, **P<0.01, ***P<0.001 (unpaired Student’s *t*-test analysis between two indicated groups).

In GPR56-KD cells, basal viability at 5 mM glucose was already reduced (Figure 4B). In this setting, dapagliflozin had no effect, peptides showed blunted effects, and glibenclamide further reduced viability. At 25 mM glucose, neither dapagliflozin nor peptides rescued viability, while glibenclamide aggravated the reduction.

### Peptides prevent cytokine-induced reduction in β-cell viability

EndoC-βH1 cells were cultured for 24 h with or without the cytokine cocktail (IL-1β: 1 ng/ml, TNF-α: 50 ng/ml, and IFN-γ: 50 ng/ml), in the presence of dapagliflozin (10 μM), peptides, or glibenclamide (5 μM). As shown in Figure 4C, cytokine exposure markedly decreased cell viability. Dapagliflozin did not affect the reduced cell viability induced by cytokines, whereas the peptide mixture fully prevented it, while glibenclamide further aggravated cytokine-induced cytotoxicity.

### Peptides increase liver cell viability in a GPR56-dependent manner

Since a recent finding has shown that GPR56 activation prevents pyroptosis and fibrosis in liver tissue [34], we next tested our peptides' effects on hepatic cells. HepG2 and Hep1–6 cells were cultured for 24 h at 5 mM glucose with various peptide concentrations in scrambled control and GPR56-KD conditions.

As shown in Supplementary Figure S3A, peptides increased HepG2 viability in a dose-dependent manner, and GPR56-KD abolished this effect. Similarly, in Hep1–6 cells, peptides dose-dependently increased cell viability, and this effect was markedly reduced after GPR56-KD (Supplementary Figure S3B).

## Discussion

GPR56 (ADGRG1) belongs to the adhesion GPCR family and has been identified as one of the most abundantly expressed GPCRs in human and mouse pancreatic islets. Our earlier profiling work demonstrated that GPR56 is the predominant GPCR in β-cells, making it of particular interest for pharmacological investigation and therapeutic exploration [7,10,22]. Several endogenous and exogenous ligands have been described, including Coll III, tissue transglutaminase, heparin, and the synthetic Stachel peptide P19, along with small molecule agonists such as L-phenylalanine, 3-α-acetoxydihydrodeoxygedunin [35], gedunin, and khivorin [18]. Among these, Coll III remains the most potent and well-studied activator of GPR56, but its large molecular size limits its practical utility for pharmacological or clinical applications.

Although the direct therapeutic use of Coll III remains limited due to its structural complexity and large molecular size, its interaction with GPR56 and the subsequent activation of intracellular signaling cascades are well established, albeit not yet completely elucidated [10–13]. This underscores the need for developing short Coll III-derived peptides as potential selective agonists of GPR56.

To address this limitation, we designed three complementary short peptides based on Coll III DNA sequences with potential binding relevance for GPR56 activation. Importantly, we used GPR56-KD cells as an internal control to validate receptor specificity. Since knockdown directly diminishes receptor-mediated cellular responses, it provides a straightforward and accurate way to ensure that observed effects are indeed GPR56-dependent. In our study, the absence of effects from the peptide mixture in GPR56-KD models strongly supports the conclusion that the biological actions of these peptides, such as enhancing cellular cAMP levels and potentiation of GSIS, as well as increased cell viability, are mediated via the GPR56-dependent pathway.

Although we did not perform direct biochemical stability analyses, the biological activity of the peptides over extended incubation/culture periods provides functional evidence of their sufficient stability under our experimental conditions. Each peptide mixture produced reproducible effects on cAMP generation, GSIS, and β-cell viability not only during short incubation times (60 min) but also after 24–72 h of continuous exposure. Such sustained activity indicates that the sequences of peptides remain intact long enough to activate GPR56 and elicit downstream signaling. In support of this notion is the fact that peptides containing N-terminal or C-terminal protection (e.g., amidation) and cysteine residues are less susceptible to rapid proteolytic degradation in aqueous environments [36], which is consistent with the prolonged functional responses that we observed with our peptides. While these findings imply adequate *in vitro* stability, formal pharmacokinetic and degradation analyses will be required to determine their half-life and metabolic fate for physiological systems *in vivo*.

Another noteworthy feature of our findings is the cooperative nature of our peptides, while individually they did not exert any effect, combinations of two or three peptides consistently triggered GPR56-dependent responses. Although we did not perform structural modeling, one possible mechanistic explanation could be that the short sequences contain complementary motifs that, when present together, recreate a minimal binding interface analogous to regions within collagen III. This could allow the peptides to bind multiple contact points on GPR56 simultaneously, consistent with the receptor’s requirement for multivalent or spatially organized ligands [13,14]. An alternative possibility could be that the peptides stabilize each other conformationally or enhance local peptide concentration near the receptor surface, thereby facilitating activation. These explanations remain hypothetical at this stage but provide a basis for future receptor-ligand modeling and biophysical binding studies to characterize the structural requirements for GPR56 activation.

Taken together, these findings not only establish GPR56 as a receptor of significant translational relevance in β-cell biology but also demonstrate the feasibility of developing smaller peptide agonists that mimic Coll III activity while circumventing the pharmacological limitations of the full-length protein.

### Mechanistic insights

A central observation of our study was that the individual peptides produced little or no detectable effect on cell viability under the conditions examined, whereas double and triple combinations of the peptides produced enhanced responses. This concerted effect of the peptides indicates a cooperative mode of action, suggesting that two or more peptide fragments are required to generate a functional GPR56-dependent effect.

Elevation of cAMP is a well-established pathway for incretin-mediated insulin secretion, with hormones such as GLP-1, GIP, and PACAP acting via their specific GPCRs to augment GSIS [23,28,32,33]. Since in the current study, cAMP generation and insulin secretion were evaluated using the peptide mixture rather than each individual peptide and combination separately, the present data do not allow us to determine whether the combined effects represent pharmacological synergy, additivity, or another form of cooperative interaction. Our findings that the peptide-induced cAMP elevation was abolished in GPR56-KD cells demonstrate that the effect of peptide mixture at least in part, is GPR56-dependent. Moreover, since no direct receptor-binding or structural studies were performed, the possible explanations involving simultaneous receptor interactions, conformational stabilization, or formation of a composite binding interface for our peptides remain speculative. Dedicated concentration–response and GPR56-binding studies will be required to determine the molecular basis of the enhanced response to peptide combinations.

In addition to promoting insulin secretion, peptide combinations also increased β-cell viability under metabolic or inflammatory stress conditions. Exposure of β-cells to pro-inflammatory cytokines (IL-1β, TNFα, and IFNγ) typically reduces cell viability and impairs insulin secretion, mimicking the diabetogenic environment [10,21,37]. Here, the peptide combinations markedly preserved β-cell survival, with triple combinations showing the most significant effect. Similarly, chronic exposure to high glucose (glucotoxicity), which is associated with oxidative stress, ER stress, and mitochondrial dysfunction, diminished GSIS [24,38–40]. Remarkably, peptide treatment counteracted these deleterious effects, restoring β-cell functionality to near-normal levels. It tempts to speculate that the cytoprotective effects of GPR56 activation may involve modulation of stress-response pathways. Previous studies have linked cAMP signaling to β-cell survival by enhancing mitochondrial function, reducing oxidative stress, and limiting apoptosis [27,28,41–43]. It is plausible that peptide-induced GPR56 activation might enhance β-cell viability by converging on these protective mechanisms. Notably, the strong loss-of-function observed in GPR56-KD cells confirms that these protective effects are not nonspecific but rather mediated via a GPR56 signaling manner. In the current study, unrelated peptide controls (sequence-scrambled) were not included. Therefore, although the attenuation of the peptide-induced effects following GPR56-KD supports a role for GPR56, the present experiments do not fully exclude sequence independence or nonspecific effects of the peptide combinations. Future studies incorporating appropriately designed sequence-scrambled controls will further establish the sequence specificity of these responses.

### Comparisons and translational implications

To place the biological activity of our peptides into a pharmacological context, we compared their effects on β-cell viability with two clinically established anti-diabetic agents, i.e. dapagliflozin and glibenclamide, with different modes of action [44–47] to illustrate the possible differences in cellular responses rather than to provide therapeutic benchmarks. The comparison revealed striking differences in mechanism and outcome. Dapagliflozin, while effective as reported, in lowering plasma glucose *in vivo* through renal glucose excretion [45], exerted no appreciable protective effect on β-cell viability in our experimental system. On the other hand, glibenclamide, which stimulates insulin release by closing K^+^_ATP_ channels [48], consistently reduced β-cell viability under both basal and stressed culture conditions. These findings align with clinical evidence suggesting that sulfonylureas, although effective in the short term [44,49], may accelerate β-cell exhaustion and loss of function, as we also reported earlier [37]. In contrast, our peptide combinations elicited cAMP-dependent protective effects in a GPR56-dependent manner. The approaches therefore show mechanistic contrasts, not efficacy comparisons, and highlight how the GPR56-mediated signaling pathway differs fundamentally from these established antidiabetic drug pathways.

Our peptide combinations, by comparison, not only potentiated insulin secretion in a glucose- and receptor-dependent manner but also provided significantly increased β-cell viability. Importantly, their activity was abolished in GPR56-KD cells, confirming that the peptides act through a GPR56-dependent manner rather than via off-target effects. These dual profiles enhancing GSIS while simultaneously preserving cell survival speak in favor of the peptide mixture as mechanistically distinct from currently used sulfonylureas like glibenclamide, which exert unbeneficial effects on β-cells [37]. Thus, these findings provide an *in vitro* basis for further investigation of the biological properties and therapeutic potential of the peptide combinations.

Beyond pancreatic islets, our results extend the potential relevance of GPR56 activation to hepatic tissue. Both HepG-2 and Hep1–6 cells displayed concentration-dependent increases in viability when exposed to the peptide mixture, an effect that was again lost after GPR56 knockdown. These observations are noteworthy, as the liver is central to glucose metabolism and systemic energy homeostasis [50,51]. We have previously reported that GPR56 is highly expressed in the liver [10] and also shown by others that GPR56 functions as a repressor of pyroptosis, thereby preventing fibrosis [34]. The ability of our peptide combinations to promote hepatocyte survival is consistent with a broader role for GPR56 in maintaining metabolic tissue integrity.

The combined β-cell and hepatocyte findings suggest that the GPR56-associated effect of our three short peptides may warrant further investigation in models of metabolic dysfunction for the simultaneous improvement of insulin secretion and liver health. This dual action is particularly relevant in T2D, where both β-cell dysfunction and dysregulation of hepatic metabolism drive disease progression [1,50–52]. Notably, the receptor-dependent nature of these effects, as confirmed by GPR56-KD experiments, provides strong mechanistic support and minimizes the likelihood that these peptides act via nonspecific pathways.

### Findings, limitations, and future directions

Taken together, the present study identifies and characterizes three short peptides derived from DNA strand alignments of Coll III with GPR56, which in combination selectively activate GPR56 and exert protective effects on pancreatic β-cells, as well as improve hepatocyte viability. Unlike conventional agonists of GPR56, such as Coll III [10,13] or synthetic Stachel peptides [53], these novel peptides are smaller, synthetically accessible, and functionally active only in synergistic combinations, i.e. double or triple combinations. This unique mode of action is highly desirable from a pharmacological perspective, as it provides receptor specificity while minimizing the risk of off-target stimulation.

## Conclusion

In conclusion, we report the discovery of short synergistic peptides that activate GPR56, potentiate β-cell function, and protect both pancreatic and hepatic cells from metabolic and inflammatory stress. These findings broaden our understanding of GPR56 biology and validation of an ‘*in vitro’* proof of concept that might be developed to a potential starting point for the development of GPR56-targeted peptide-based approaches, pending further mechanistic and *in vivo* validation.

## Future direction

While our findings reveal a reproducible and receptor-specific biological effect of the peptide combinations, pharmacokinetic properties such as peptide half-life, degradation, and bioavailability as well as computational modeling and whole animal testing need to be examined in future studies to provide definitive proof of the translational applicability of these peptides.

## Supplementary Material

Supplementary Figures S1-S3 and Tables S1-S2

## Data Availability

The information on the sequences of designed peptides and primers is found in Supplementary Figure S2 and Supplementary Tables S1 and S2. All other data is available to non-profit organizations upon request at Lund University, Lund, Sweden: lu@lu.se.

## Open Access

Open access for this article was enabled by the participation of Lund University in an all-inclusive Read & Publish agreement with Portland Press and the Biochemical Society under an agreement with BIBSAM.

## Abbreviations

Coll III: collagen type III
HEPES: N-2 hydroxyethylpiperazine-N′-2-ethanesulfonic acid
GPCRs: G-protein coupled receptors
GPR56: G protein-coupled receptor 56
GPR56-KD: GPR56 knockdown
GSIS: glucose-stimulated insulin secretion
qPCR: quantitative polymerase chain reaction
T2D: type 2 diabetes
