# Supplementary material for: Design of short-sequenced collagen type III-derived peptides with GPR56 dependent biological activity
Source: Biosci Rep. 2026 Nov 3;46(11):BSR20250320. doi: 10.1042/BSR20250320 (PMC13741089; doi:10.1042/BSR20250320)
Supplement: Supplementary file 1 [file BSR-2025-0320_supp.pdf]

Supplementary Tables

Design of short-sequenced collagen type III-derived peptides with GPR56 dependent biological activity

Israa Mohammad Al-Amily[1*], Fariborz Parandeh[1*], Pontus Dunér[2], Marie Sjögren, and Albert Salehi[1].

Department of Clinical Science, Malmö, Division of Islet Cell Physiology[1] and Experimental cardiovascular research[2], University of Lund, Sweden

Table S1. The list and identities of siRNA and shRNA used in the study.

GPR56 shRNA lentivirus (m)	Santa Cruz	sc-60750
GPR56 (ADGRG1) siRNA (r)	ThermoFisher Scientific	s141373
GPR56 (ADGRG1) siRNA (r)	ThermoFisher Scientific	s141374
GPR56 (ADGRG1) siRNA (r)	ThermoFisher Scientific	s141375
GPR56 (ADGRG1) siRNA (h)	ThermoFisher Scientific	s17757
GPR56 (ADGRG1) siRNA (h)	ThermoFisher Scientific	s17759
GPR56 (ADGRG1) siRNA (h)	ThermoFisher Scientific	s17758

Design of short-sequenced collagen type III-derived peptides with GPR56 dependent biological activity

Israa Mohammad Al-Amily[1*], Fariborz Parandeh[1*], Pontus Dunér[2], Marie Sjögren and Albert Salehi[1]

Department of Clinical Science, Malmö, Division of Islet Cell Physiology[1] and Experimental cardiovascular research[2], University of Lund, Sweden

Table S2. Primer sequence identities used in the study.

GPR56 primer (human)	AB applied biosystem	Hs00608272_m1
GPR56 primer (rat)	AB applied biosystem	Rn01131883_gH
GPR56 primer (mouse)	AB applied biosystem	Mm00726417_s1
HPRT primer (human)	ThermoFisher Scientific	Hs01003267_m1
PPIA primer (human)	AB applied biosystem	Hs04194521_s1
HPRT primer (rat)	AB applied biosystem	Rn01527840_m1
PPIA primer (rat)	AB applied biosystem	Rn00690933_m1
HPRT primer (mouse)	AB applied biosystem	Mm00726417_s1
PPIA primer (mouse)	AB applied biosystem	Mm00726417_s1

Supplementary Figures

Design of short-sequenced collagen type III-derived peptides with GPR56 dependent biological activity

Israa Mohammad Al-Amily[1*], Fariborz Parandeh[1*], Pontus Dunér[2], Marie Sjögren[1], and Albert Salehi[1]

Department of Clinical Science, SUS, Malmö, Division of Islet Cell Physiology[1] and Experimental cardiovascular research[2], University of Lund, Sweden

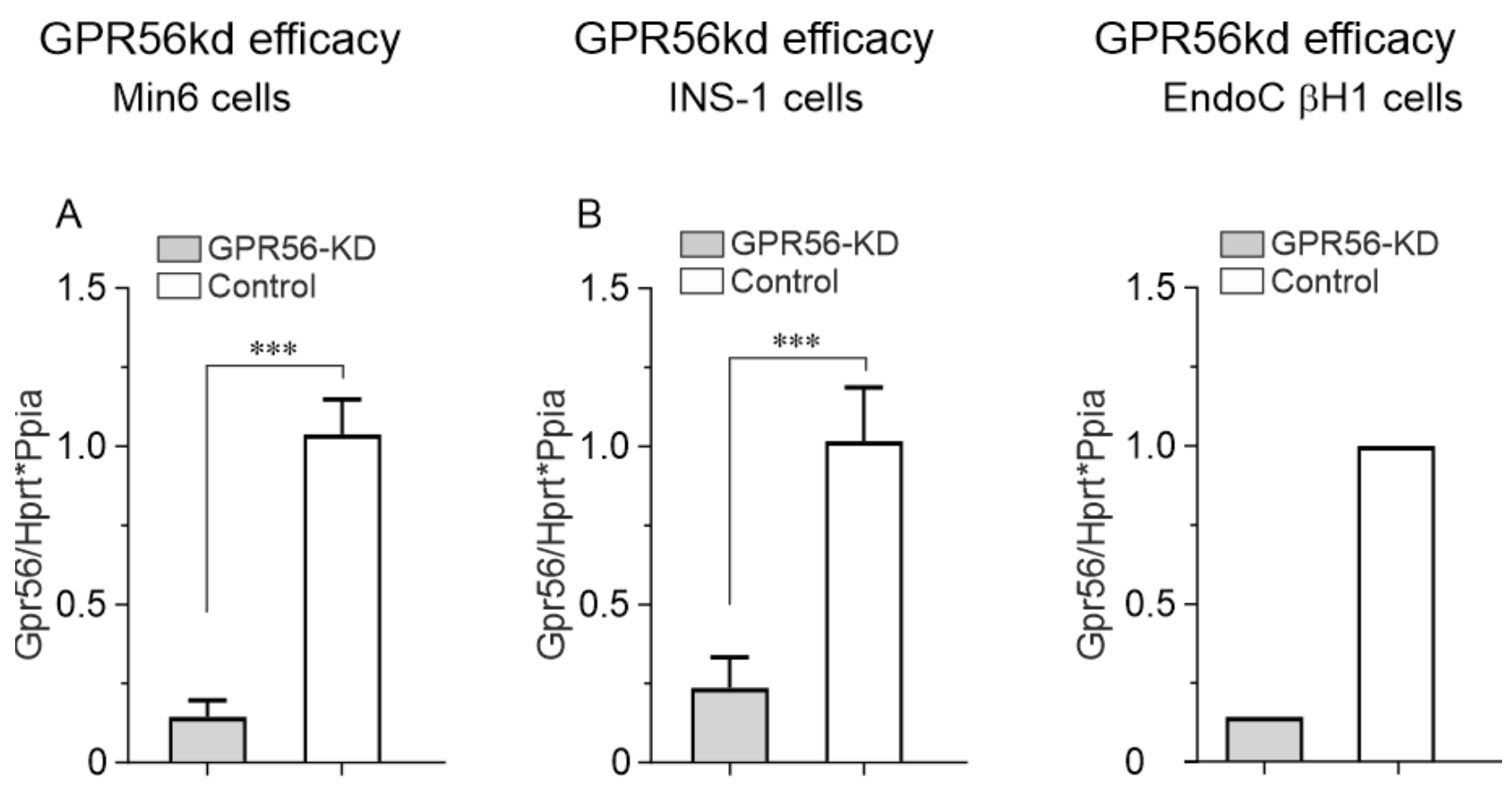

Figure S1. **The efficiency of GPR56-KD in Mins6, INS-1 832/13 and EndoC βH1 cells** Analysis of GPR56 mRNA by qPCR showing a reduced expression in GPR56-KD (internal control of KD efficiency) compared to scramble controls cells. Mean±SEM from n=6 (Min6 cells), n=4 (INS-1 832/13 cells) and EndoC βH1 cells is shown. ***p <0.001 (unpaired *Student´s* t-test analysis).

A

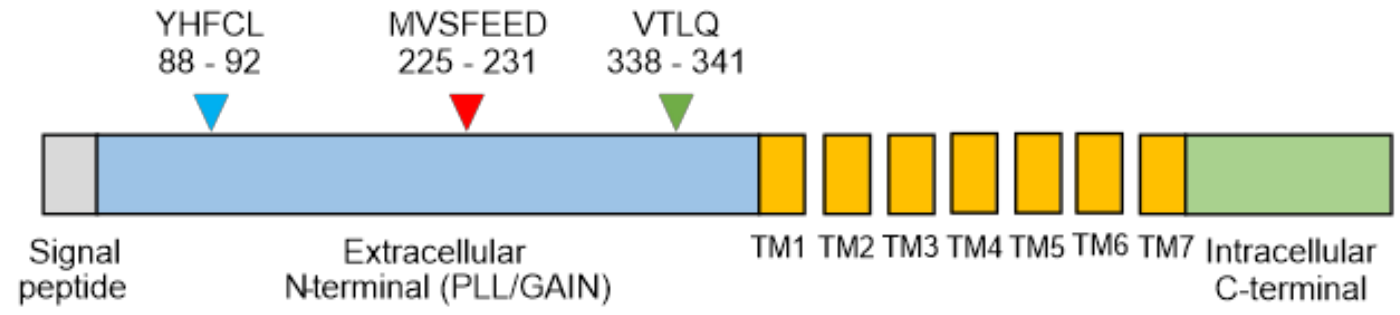

B

aa (nr)	
1-90	MTPQSLLQTTLFLLSLLFLVQGAHGRGHREDFRFCSQRNQTHRSSLHYKPTPDLRISIENSEEALTVHAPFPAAHPASRSFPDPRGYHF
91-180	CLYWNRHAGRLHLLYGKRDFLLSDKASSLLCFQHQEESLAQGPPLLATSVTSWWSPQNISLPSAASFTFSFHSPPHTAAHNASVDMCELK
181-270	RDLQLLSQFLKHPQKASRRPSAAPASQQLQSLESKLTSVRFMGDMVSFEEDRINATVWKLQPTAGLQDLHIHSRQEEEQSEIMEYSVLLP
271-360	RTLFQRTKGRSGEAEKRLLLVDFSSQALFQDKNSSQVLGEKVLGIVVQNTKVANLTEPVVLTFQHQLQPKNVTLQCVFWVEDPTLSSPGH
361-450	WSSAGCETVRRETQTSCFCNHLTYFAVLMVSSVEVDAVHKHYLSLLSYVGCVVSALACLVTIAAYLCSRVPLPCRRKPRDYTIKVHMNLL
451-540	LAVFLLDTSFLLSEPVALTGSEAGCRASAIFLHFSLLTCLSWMGLEGYNLYRLVVEVFGTYVPGYLLKLSAMGWGFPIFLVTLVALVDVD
541-630	NYGPIILAVHRTPEGVIYPSMCWIRDSLVSYITNLGLFSLVFLFNMAMLATMVVQILRLRPHTQKWSHVLTLLGLSLVLGLPWALIFFSF
631-693	ASGTFQLVVLYLFSIITSFQGFLIFIWYWSMRLQARGGPSPLKSNSDSARLPISSGSTSSSRI

C

Peptide 1: NQGCK-NH_2 (Asn-Gln-Gly-Cys-Lys-Leu-NH_2) complementary to MVSFEED sequence of GPR56

Peptide 2: H-LLSSR-NH_2 (H-Leu-Leu-Ser-Ser-Arg-NH_2) complementary to VTLQ sequence of GPR56

Peptide 3: NH_2-GYYCG-COOH (NH_2-Gly-Tyr-Tyr-Cys-Gly-COOH) complementary with modification to YHFCL sequence of GPR56

Figure S2. **Schematic illustration of GPR56 protein structure and the targeted amino acid part for the design of small peptide agonists of GPR56**.

Schematic representation of the human GPR56 receptor protein showing the localization of N-terminal exonic part with the indicated amino acid domain binding our designed peptides and the 7 transmembrane (7 TM) regions of the GPR56 receptor as well as the intracellular C-terminal part (A). Full sequence of GPR56 amino acid sequences and the indicated (red marked) amino acid sequences that is targeted by the presented peptide agonists shown in (C). The amino acid sequence of three designed peptides based on Coll III protein tested in the present study (C). The last two amino acids of peptide 3 were modified and replaced by -CG- to strengthen the peptide stability (see also table I).

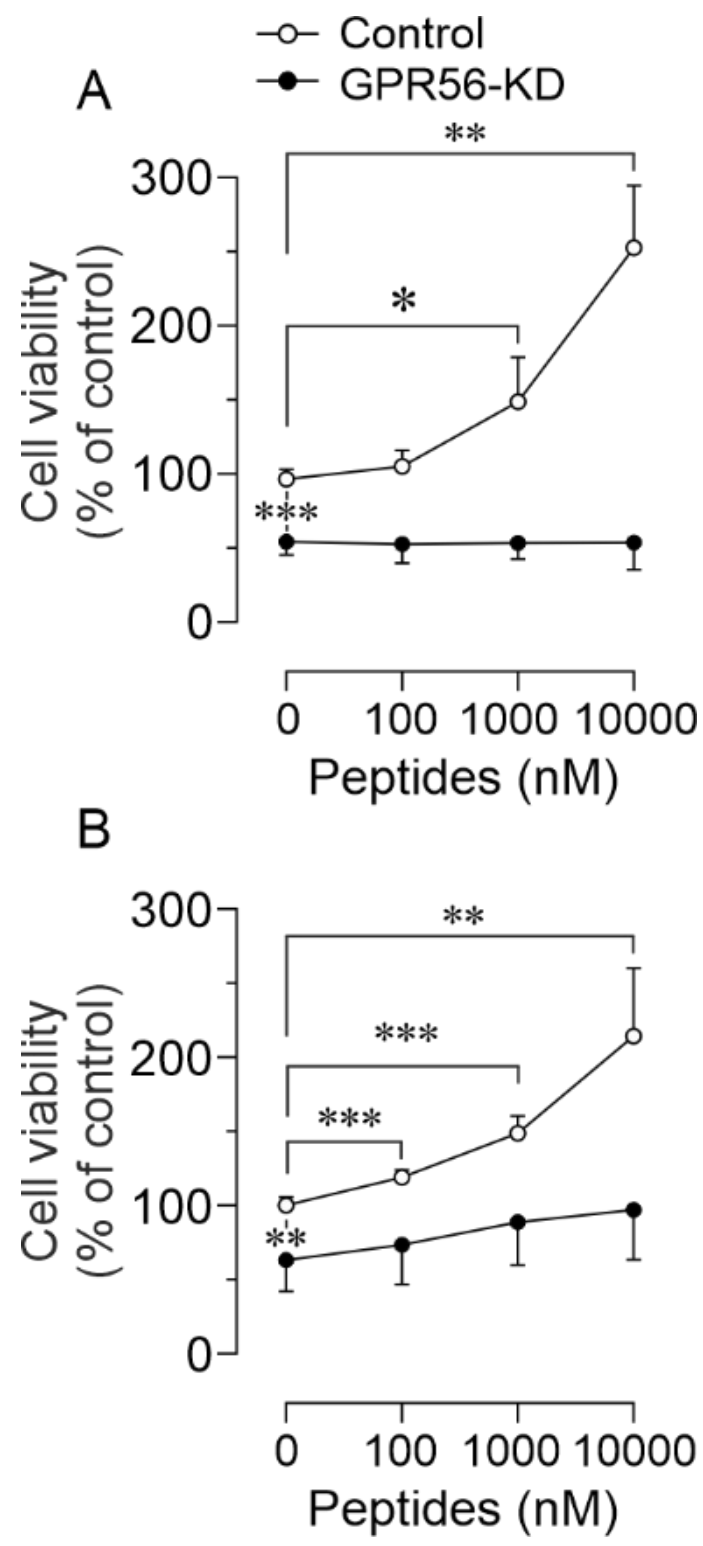

Figure S3. The impact of GPR56 expression on liver cell functionality in human and mouse liver cell line

Scrambled control and GPR56-KD human HepG-2 cells cultured with different concentration of peptide-mixture for 24h showed a concentration-dependent increase in cell viability (A). GPR56-KD decreased cell viability and also abolished in the peptides effect. Mean ± SD for n=4 observations performed at different occasions are shown. *p<0.05, **p < 0.01 (unpaired *Student´s* t-test analysis). (B) The concentration-effect of peptide-mixture on cell viability in scrambled control and GPR56-KD mouse Hep1-6 cells cultured with different concentration of peptides for 24h. Shown is a concentration-dependent effect of peptides (B). GPR56-KD reduced the cell viability and also attenuated the effects of the peptide-mixtures. The results are mean ± SD for n=4 observations performed at different occasions. **p < 0.01, ***p<0.001 (unpaired *Student´s* t-test analysis between indicated groups).
